# Engineered Exosomes Carrying Super-Repressor IκB Reduced Biliary Atresia-Induced Liver Fibrosis in Minipig and Mouse Models

**DOI:** 10.3390/pharmaceutics17020264

**Published:** 2025-02-17

**Authors:** Jisoo Kang, Cheolhyoung Park, Hanoul Yun, Chulhee Choi, Wonhyo Seo

**Affiliations:** 1 College of Pharmacy, Graduate School of Pharmaceutical Sciences, Ewha Womans University, Seoul 03760, Republic of Korea; 2 ILIAS Biologics Inc., Daejeon 34014, Republic of Korea; chpark@iliasbio.com (C.P.); hyun@iliasbio.com (H.Y.); 3 Gradutate Program in Innovative Biomaterials Convergence, Ewha Womans University, Seoul 03760, Republic of Korea

**Keywords:** biliary atresia, liver fibrosis, engineered exosome, super-repressor IκB

## Abstract

**Background and Aim:** Biliary atresia is a rare, progressive disease that affects the bile ducts in newborns. Persistent bile duct obstruction induces various pathological conditions, including jaundice, inflammation, and liver fibrosis; however, the exact pathogenesis of biliary atresia is not yet fully understood. Nuclear factor-κB (NF-κB) is widely acknowledged as a key regulator in the pathogenesis of hepatitis and liver fibrosis, and extensive research has been conducted to develop strategies to effectively inhibit its activity to mitigate liver damage. Exosome-based therapeutic platforms offer targeted NF-κB inhibition with low immunogenicity and enhanced liver-specific delivery. This study aimed to evaluate the therapeutic efficacy of Exo-SrIκB in treating cholestatic liver fibrosis using experimental animal models. **Methods:** Exo-SrIκB (an exosome-based therapy containing the super-repressor IκB protein) using EXPLOR technology (Exosome engineering for Protein Loading via Optically Reversible protein-protein interactions) to encapsulate the super repressor IκB (SrIκB) within exosomes. The therapeutic efficacy of Exo-SrIκB was assessed in minipig and mouse models with experimentally induced cholestatic liver disease. **Results:** Administration of Exo-SrIκB significantly attenuated liver fibrosis progression in both animal models by inhibiting NF-κB nuclear translocation and reducing the expression of fibrotic markers. Treated animals exhibited reduced collagen deposition, lower α-SMA levels, and improved hepatic function compared to untreated controls. **Conclusion:** Exo-SrIκB effectively suppressed NF-κB signaling and alleviated liver fibrosis in experimental cholestatic liver disease models, suggesting that exosome-based therapeutics may offer a targeted and biocompatible application to managing liver fibrosis and other chronic liver diseases.

## 1. Introduction

Cholestatic liver disease can arise from a range of conditions, which include exposure to toxins, infection, bile flow blockages, gut microbiota imbalances, and genetic deficiencies. These factors impair bile flow by disrupting bile production or excretion in the liver, which resulted in the accumulation of toxic bile acids within hepatocytes and/or the circulatory system [1,2,3]. Clinically, cholestasis leads to the retention of bile constituents in the bloodstream, and is classified as either intrahepatic or extrahepatic, depending on the site of bile flow obstruction [4]. Biliary atresia, the most common cause of obstructive jaundice, is a progressive fibro-inflammatory disorder that affects part or all of the extrahepatic biliary tree, ultimately resulting in the obliteration of the affected bile ducts. This condition is characterized by progressive fibro-obliteration and destruction of the intrahepatic and/or extrahepatic bile ducts during the neonatal period [5,6]. Portoenterostomy, a surgical procedure in which the damaged bile ducts are removed and the small intestine is surgically connected to the liver, is the primary therapeutic approach for the treatment of biliary atresia [7]. While this procedure significantly improves short-term outcomes, the majority of patients still need a liver transplant for sustained long-term survival [8]. Both genetic and environmental influences have been identified as potential contributors to the worsening of biliary atresia; however, its underlying pathogenesis remains unclear. Liver fibrosis is a key feature of biliary atresia, and it progresses more rapidly in biliary atresia than in any other hepatic or biliary injury [9,10].

Nuclear factor-κB (NF-κB) is a heterodimeric transcription factor that is critically involved in the development of various liver diseases, such as hepatitis and fibrosis [11]. Previous studies have highlighted significant changes in NF-κB expression in the liver tissues of biliary atresia patients, as well as in a mouse model subjected to bile duct ligation [12]. In addition, administering NF-κB inhibitors can effectively suppress liver fibrosis progression by downregulating key fibrotic markers, including collagen I and α-SMA [13]. These findings strongly indicate that NF-κB inhibition could serve as a promising therapeutic strategy for managing chronic diseases, including liver fibrosis. Despite substantial efforts to develop NF-κB inhibitors for clinical use, significant challenges have hindered their therapeutic application. A primary obstacle is the lack of specificity in traditional NF-κB inhibitors, which frequently act on multiple signaling pathways, resulting in unintended side effects and toxicity [14,15]. In response to these challenges, there is an urgent need for innovative drug delivery systems that prioritize selective and potent NF-κB inhibition while minimizing immune activation and off-target effects.

The secretion of cell-derived extracellular vesicles and exosomes has been recognized as a physiological process by which they serve as carriers of biological components, such as mRNA, miRNA, proteins, and lipids. These cell-oriented vesicles can reflect the condition of the host cells [16,17]. Due to their low immunogenicity and biocompatibility, exosomes are capable of delivering biomolecules to target cells without degradation of RNA or loss of biological information. Therefore, cell-derived exosomes have been suggested as a novel drug delivery platform for gene therapy and chemotherapy, highlighting their clinical significance [18]. Previous research demonstrated that exogenously administered exosomes were predominantly concentrated in the liver and interact with various hepatic parenchymal/non-parenchymal cells to regulate hepatic homeostasis. Given these characteristics, recent research has focused on the use of exosomes to mitigate various liver diseases, including liver fibrosis [19,20,21,22,23,24,25]. Exo-SrIκB is an exosome-based therapeutic platform designed to deliver a super repressor IκB (SrIκB), a dominant-active mutant form of the IκB protein. This mutant form is able to effectively inhibit NF-κB signaling and is encapsulated into exosomes through optically reversible protein–protein interaction (EXPLOR) technology, enabling precise control over its loading and delivery. EXPLOR delivers cargo proteins freely into the cytosol. Cargo proteins and tetraspanin (e.g., CD9) interact via fusion with cytochrome 2 (CRY2) and a truncated domain of calcium- and integrin-binding protein 1 (CIBN), respectively. CRY2 and CIBN bind under blue light exposure and dissociate in its absence [26]. Exo-SrIκB has been applied to treat a variety of inflammatory disorders, including acute, autoimmune, and chronic immunological diseases [27,28]. In addition, the administration of Exo-SrIκB has been shown consistently to reduce the progression of hepatic inflammation in alcohol-associated liver disease by inhibiting NF-κB nuclear translocation [28]. To investigate the potential of Exo-SrIκB as a novel drug delivery system, this study aimed to assess its therapeutic potential in cholestatic liver disease-related fibrosis by applying it to minipig and mouse models with experimentally induced cholestatic disease.

## 2. Methods

### 2.1. Animals

Fourteen-month-old female minipigs (*Sus scrofa*), each weighing approximately 24 kg, were purchased from CRONEX Co., Ltd. (Cheongju, Republic of Korea), and the animal experiments were performed at HLB BioSTEP Co., Ltd. (Incheon, Republic of Korea). The animals were kept in specific pathogen-free (SPF) animal facilities with complete substrate feeding, according to the standard guidelines for laboratory animals. Prior to the experiments, the animals underwent a one-week quarantine and acclimatization period under controlled environmental conditions, maintained at a temperature of 23 ± 3 °C and a humidity of 55 ± 15%. Each minipig was identified using an ear tag and subjected to a 24-h fasting period before undergoing radiofrequency ablation (RFA)-induced bile duct stricture. For the bile duct ligation (BDL) mouse model, 12-week-old male C57BL/6N mice (Koatech, Ansan-si, Republic of Korea) were purchased and acclimated to the same environment. The mice were housed in a temperature- and humidity-controlled facility with a 12-h light/dark cycle and were provided ad libitum access to food and water. All animal experiments were performed according to a protocol approved by the Institutional Animal Care and Use Committee at the National Center of Efficacy Evaluation for the Development of Health Products Targeting Digestive Disorders (NCEED) and Ewha Womans University (EWHA IACUC 23-002-2, approval date: 5 January 2024).

### 2.2. Radiofrequency Ablation (RFA)-Induced Biliary Stricture in a Porcine Model

Endobiliary radiofrequency ablation (EB-RFA)-guided endoscopic retrograde cholangiography was performed using an RFA catheter with temperature control. An endobiliary radiofrequency catheter (ELRA™ RF catheter, STARmed, Goyang, Republic of Korea) and a power RFA generator (VIVA Combo™; STARmed, Goyang, Republic of Korea) were used for EB-RFA. Radiofrequency energy was delivered by an RFA generator, operating at 480 kHz, in target temperature-controlled mode (80 °C, 7 W for 90 s). At 3 weeks after RFA-induced biliary stricture, a 4 cm NEXENT biliary stent was inserted into the EB-RFA to release the pressure from the blocked biliary tract, and euthanasia for autopsy was performed 5 weeks after EB-RFA induction [29,30]. One minipig was anesthetized without EB-RFA and served as the normal control.

### 2.3. Evaluation of Body and Liver Weights of Minipigs

Body weight was measured once a week throughout the experiments. The body weight of each subject was also measured prior to performing EB-RFA, immediately before administering Exo-SrIκB, and at the time of autopsy. The miniature pigs were anesthetized, and blood was drawn from the vein, followed by exsanguination at the end of the study. The weight of the collected whole-liver tissue was measured, and the livers were photographed. To minimize measurement errors, the body weights of the subjects were consistently measured using the same scale.

### 2.4. Bile Duct Ligation-Induced Liver Fibrosis Mouse Model

For the induction of acute cholestasis, the BDL mouse model was applied in this study [31]. To prevent postoperative sepsis, drinking water containing antibiotics (Enrofloxacin: 250 μg/mL) was administered. Mice were anesthetized by inhalation of 4 vol% isoflurane in 100% oxygen at a flow rate of 4 L/min. Subsequently, the peritoneal cavity was surgically opened, and the common bile duct was double-ligated [32].

### 2.5. Production of Exo-SrIκB

Exosome production proceeded as described previously [27,33]. In brief, genetically modified Expi293F^TM^-producing cells were incubated for four days in a wave culture system, and the cells were exposed to blue-light illumination for target protein loading and exosome production. After incubation, the culture medium was harvested and centrifuged at 2000× *g* for 10 min to remove cells and debris. A 0.22 µm polyethersulfone filter was used to remove any large-sized particles. To reduce the risk of loading the SEC column (Cytiva, Marlborough, MA, USA) with impurities that would exceed its binding capacity, the samples were subjected to diafiltration and concentration through TFF (Tangential flow filtration, Sartorius AG, Göttingen, Germany). After TFF, the concentrated medium was loaded onto an SEC column for further purification. A second TFF was then performed to concentrate the exosomes.

### 2.6. Administration of Exo-SrIκB to the Animal Models

For the minipig models, the Exo-SrIκB dosage was calculated based on body weight (1.6 × 10^10^ particles/kg) and administered intravenously for 1 h using an infusion pump at 1, 2, 3, and 4 weeks after the induction of biliary RFA. Phosphate-buffered saline (PBS; 1 mL/kg) was administered to the control group using the same method. In the mouse model, Exo-SrIκB was injected twice (on day 0 and day 5) via the intravascular route over the 11 days required to induce BDL-induced liver fibrosis. Alternatively, Exo-SrIκB was administered intraperitoneally 8 times over 21 days in the BDL-induced fibrosis group. The same quantity of PBS or naïve exosome was administered to the control groups.

### 2.7. Blood Cell Count and Serum Analyses

After collecting blood from the minipigs and mice, whole blood count analysis was performed. Serum samples were further collected after high-speed centrifugation, and blood biochemistry testing was conducted using an automatic blood biochemical analysis device (7180 Hitachi, Tokyo, Japan, and Fujifilm DRI-CHEM-NX500, Tokyo, Japan) according to the manufacturer’s instructions.

### 2.8. Histological Analysis

After completing the experiment, the weight of the extracted liver tissue from each minipig was measured to identify the changes in the relative weight of the liver, and the morphological changes were also recorded. Parts of the left and median lobes of the mouse liver were fixed with 10% neutral buffered formalin. After deparaffinization and rehydration, paraffin sections of 4 μm thicknesses were subjected to H&E, Masson’s trichrome, and Sirius red staining. The extent of liver inflammation and fibrosis, piecemeal necrosis, and lobular necrosis were evaluated by a pathologist, and histological activity and fibrosis scores were obtained according to the criteria listed in a previous study [34].

### 2.9. RNA Extraction and Quantitative Real-Time PCR

For the qRT-PCR assays, total RNA was extracted from liver tissue samples of each animal using the TRIzol™ reagent (#15596018; Invitrogen, Waltham, MA, USA). The RNA concentrations were measured, and complementary DNA (cDNA) was synthesized using the High-Capacity cDNA Reverse Transcription Kit (Applied Biosystems, Waltham, MA, USA) following the manufacturer’s protocol. qRT-PCR was conducted using the SYBR Green Real-Time PCR Master Mix (TOYOBO, Osaka, Japan). The amplification reactions were performed on the Biorad CFX96™ Real-Time PCR Detection System at the Ewha Drug Development Research Core Center. Gene expression levels were analyzed using the ΔΔCT method, with target gene mRNA expression normalized to 18S rRNA expression.

### 2.10. Western Blot Analysis

Liver tissue lysates from mice were homogenized in RIPA lysis buffer at 4 °C and centrifuged at 10,000× *g* for 10 min. Nuclear and cytoplasmic fractions were isolated using NE-PER^TM^ Nuclear and Cytoplasmic Extraction Reagents (#78833; Thermo Fisher Scientific, Waltham, MA, USA). Following tissue homogenization, the supernatants were combined with loading buffer and subjected to SDS-PAGE. Protein lysates were separated on 4–12% triglycine gradient gels (Criterion XT, BioRad, Hercules, CA, USA) and subsequently transferred onto nitrocellulose membranes. The membranes were then blocked with 5% bovine serum albumin and incubated with primary antibodies at a 1:1000 dilution in PBST. Protein bands were detected using the SuperSignal West Femto Maximum Sensitivity Substrate (#34096; Thermo Fisher Scientific), and the relative protein levels were normalized to β-actin and lamin.

### 2.11. Antibodies

For the Western blot analysis, primary antibodies specific to the following proteins were utilized: α-SMA (#19245; Cell Signaling, Danvers, MA, USA), COL1A1 (#72026; Cell Signaling), phospho-NF-κB p65 (#3033; Cell Signaling), NF-κB p65 (#8242; Cell Signaling), TGF-β (#3711; Cell Signaling), 4-HNE (STA-035; Cell Biolabs), β-Actin (#4967; Cell Signaling), GAPDH (#2118; Cell Signaling), and Lamin B1 (#13435; Cell Signaling). Secondary antibodies, including HRP-linked anti-rabbit IgG (#7041; Cell Signaling) and HRP-linked anti-mouse IgG (#7076; Cell Signaling), were employed at a dilution of 1:500.

### 2.12. Statistical Analysis

The data were present as the mean ± standard deviation (SD) for the minipig model and the mean ± standard error of the mean (SEM) for the mouse model. Statistical comparisons between two or more groups were conducted using Student’s *t*-test or one-way analysis of variance (ANOVA). A *p*-value of less than 0.01 or 0.05 was considered statistically significant.

## 3. Results

### 3.1. Systemic Administration of Exo-SrIκB Effectively Protected Against the Progression of BDL-Induced Liver Fibrosis in a Mouse Model

**Figure 1:**
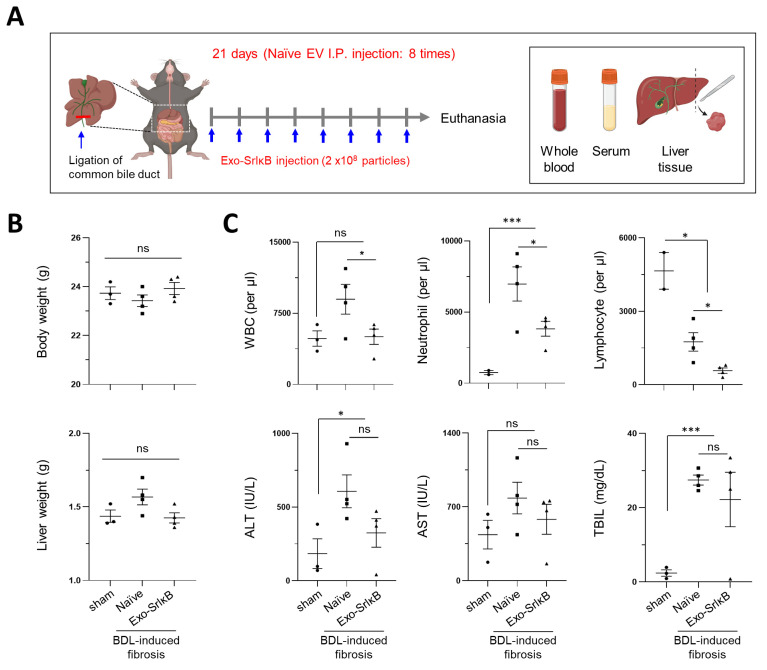
Intraperitoneal Exo-SrIκB administration improves blood cell count results and serological markers in BDL-induced liver fibrosis. (**A**) Schematic overview of the experimental timeline. (**B**) The body and liver weights were evaluated. (**C**) Whole blood and serum were subjected to complete blood counts and serum chemistry analyses. Data are expressed as the mean ± SEM. * *p* < 0.05, *** *p* < 0.001 versus the corresponding control. ns: not significant.

**Figure 2:**
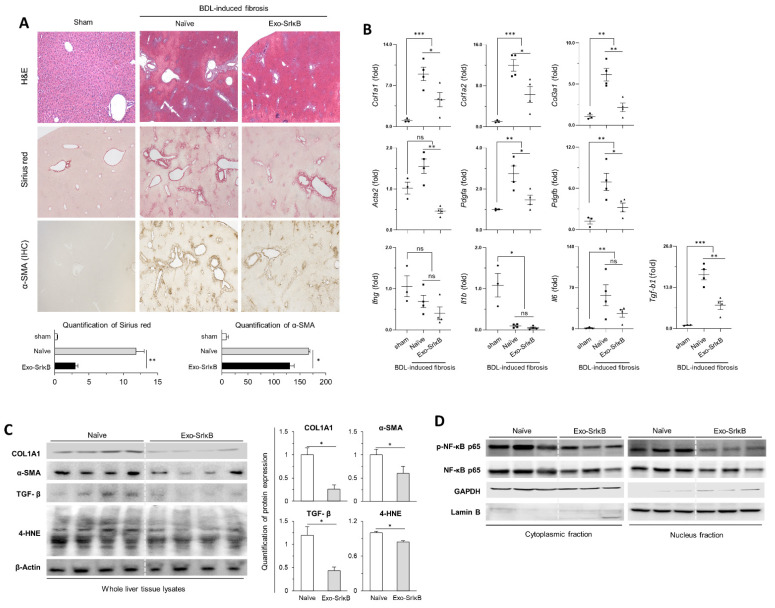
Multiple intraperitoneal Exo-SrIκB administration attenuated BDL-induced liver fibrosis in a mouse model. (**A**) Liver tissues were subjected to H&E, Sirius red, and α-SMA immunohistochemistry staining. (**B**) Liver tissues were subjected to RT-qPCR analysis. (**C**,**D**) Protein expression (liver tissue lysates and cytoplasmic/nucleus fractions) was assessed by Western blotting. Data are expressed as the mean ± SEM. * *p* < 0.05, ** *p* < 0.01, *** *p* < 0.001 versus the corresponding control. ns: not significant.

Exo-SrIκB suspension was intraperitoneally administered a total of eight times (every other day) to maximize the therapeutic effects in BDL-induced liver fibrosis (Figure 1A). Statistical significance was not observed in body weigh differences, blood cell counts, and blood chemistry analyses between the Exo-SrIκB-treated group and the control group (Figure 1B). Nonetheless, the Exo-SrIκB-treated group showed an overall decrease in the numbers of neutrophils and lymphocytes and the serum levels alanine transferase (ALT), aspartate transferase (AST), and total bilirubin compared with the control group (Figure 1C). Collagen deposition and α-SMA expression were markedly reduced in the liver sections after intraperitoneal Exo-SrIκB administration (Figure 2A), and the expressions of fibrosis- and inflammatory-associated genes were significantly lower in the whole liver tissues from Exo-SrIκB-administered mice (Figure 2B,C). Once the protein expression of NF-κB was evaluated in the extracted cytoplasmic and nuclear fractions, the expression of phospho-NF-κB p65 was lower exclusively in the nuclear fraction, not in the cytoplasmic fraction (Figure 2D).

Because the intraperitoneal administration of Exo-SrIκB significantly attenuated the progression of BDL-induced liver fibrosis, we next sought to determine whether intravenous Exo-SrIκB administration could also alleviate the progression of BDL-induced liver fibrosis. Similar to intraperitoneal administration, Exo-SrIκB (2 × 10^8^ particles/head) was administered twice intravenously (on day 0 and day 5), and the mice were euthanized at 11 days post-BDL (Supplementary Figure S1A). There were no significant differences in the body or liver weights between the two groups (Supplementary Figure S1B). The numbers of white blood cells and neutrophils were further increased in the BDL-induced liver fibrosis model compared with the sham control group. Meanwhile, the numbers of white blood cells and neutrophils were significantly lower in the Exo-SrIκB-treated group compared with the vehicle-treated group (Supplementary Figure S1C). Although statistical significance was not observed between the two groups, a decreasing tendency in the number of lymphocytes was observed in the Exo-SrIκB-treated group (Supplementary Figure S1C). The serum levels of alanine transferase (ALT), total bilirubin, alkaline phosphatase (ALP), and gamma glutamyl transferase (GGT) were comparable between the groups; however, the aspartate aminotransferase (AST) level was slightly higher in the Exo-SrIκB-treated group compared to the vehicle-treated group (Supplementary Figure S1D). Sirius Red staining and α-SMA immunohistochemistry staining clearly showed that liver fibrosis was significantly attenuated by Exo-SrIκB treatment (Supplementary Figure S2A). The increased expression of fibrosis-associated genes in the liver tissue was significantly reduced in the group treated with Exo-SrIκB. Although there was no significant difference in the expression of *Il6* and *Il1b* between the two groups, the mRNA levels of *Ifng* and *pdgfa* were lower in the Exo-SrIκB-treated group (Supplementary Figure S2B). Immunoblotting using liver tissue lysates revealed a pattern of significantly decreased fibrosis-related proteins, such as α-SMA and COL1A1, in the Exo-SrIκB-treated group. It was also confirmed that the expression of phospho-NF-κB was lower in the Exo-SrIκB-treated group (Supplementary Figure S2C left panel). As similar as the intraperitoneal route, reduced p-NF-κB p65 expression was observed in the nuclear fraction but not the cytoplasmic fraction (Supplementary Figure S2C right panel). These results clearly indicate that the systemic administration of Exo-SrIκB effectively suppressed the translocation of NF-κB to the nucleus, leading to pronounced anti-inflammatory and antifibrotic effects in the BDL-induced liver fibrosis model. Collectively, the results showed that the administration of Exo-SrIκB significantly alleviated BDL-induced liver fibrosis, regardless of the route of exosome administration.

### 3.2. Administration of Exo-SrIκB Attenuated Jaundice, Inflammation, and Liver Fibrosis in an RFA-Induced Biliary Stricture Porcine Model

**Figure 3:**
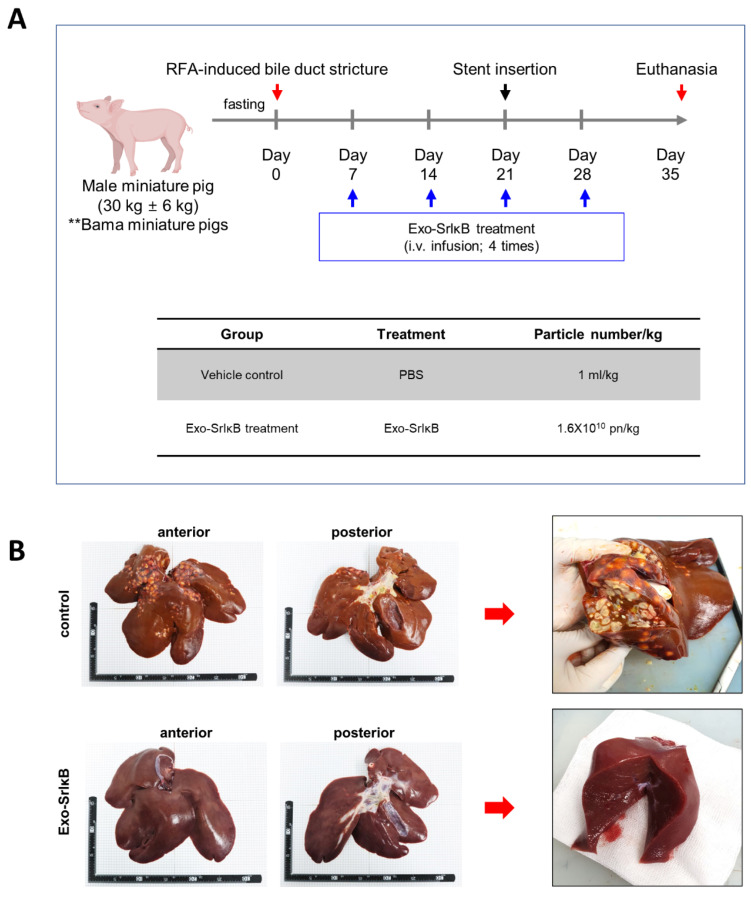
Administration of Exo-SrIκB in radiofrequency ablation (RFA)-induced biliary stricture in a porcine model. (**A**) Schematic overview of Exo-SrIκB treatment in the RFA-induced bile duct stricture model. (**B**) Gross finding of the liver tissue was assessed. **: bama miniature pigs.

To verify the therapeutic effects of Exo-SrIκB across different species, we assessed its efficacy in a porcine model of biliary atresia-induced fibrosis. Two minipig groups (vehicle control and Exo-SrIκB treatment) were organized. In the Exo-SrIκB treatment group, Exo-SrIκB (1.6 × 10^10^ particle/kg/mL) was administered intravenously on days 7, 14, 21, and 28 post-RFA-induced bile duct stricture. Then, a vascular stent was inserted on day 21 to release the biliary pressure, and the animals were euthanized on day 35. A schematic overview of this experiment is shown in Figure 3A. There was no significant body weight change throughout the experiments. In addition, the liver weight was similar between the two groups: 736.1 ± 199.6 g in the vehicle-treated group and 601.8 ± 44.6 g in the Exo-SrIκB-treated group. Although there was no statistical difference between the two groups, the relative liver weight (assessment of liver weight based on body weight) was approximately 17% lower in the Exo-SrIκB-treated group compared to the vehicle-treated group (Supplementary Figure S3A). Furthermore, we did not observe any significant differences between the two groups in the analyses of complete blood cell counts and blood chemistry at different timepoints (Supplementary Figures S3B and S4A). However, the total bilirubin levels in the serum of the animals in the Exo-SrIκB-treated group was approximately 2.4 times lower than that in the vehicle-treated group at the point of sacrifice (Supplementary Figure S3B).

**Figure 4:**
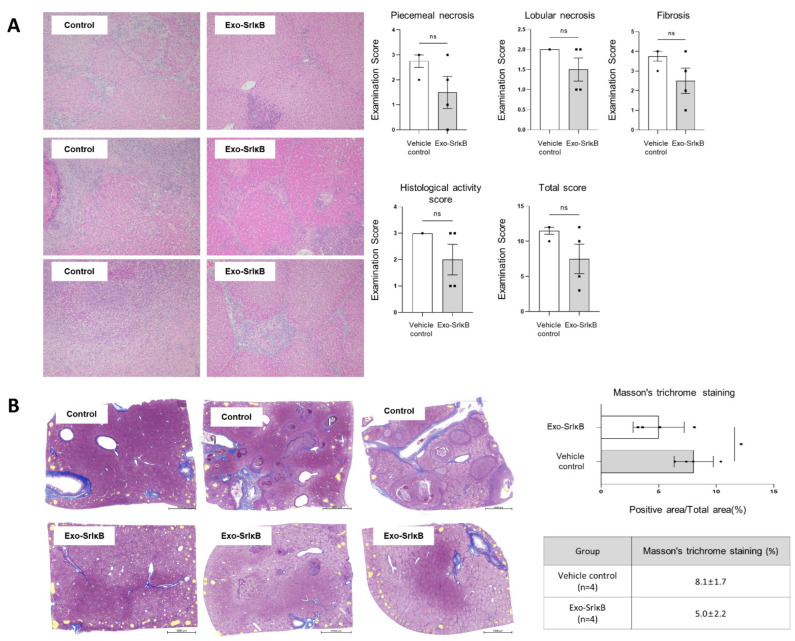
Administration of Exo-SrIκB mitigates hepatic functions and liver fibrosis in RFA-induced biliary stricture porcine model. (**A**,**B**) Liver tissues were subjected to H&E and Masson’s trichrome staining (scale bars = 5000 μm). The levels of piecemeal necrosis, lobular necrosis, and histological activity were scored by a pathologist. Data are expressed as the mean ± SD. * *p* < 0.05 versus the corresponding control. ns: not significant.

As a clinical symptom of biliary atresia, jaundice was observed in the gums and sclera of the vehicle control group during the experiments, whereas it was noticeably less pronounced in the Exo-SrIκB-treated group. Purulent inflammation in the liver was observed in the vehicle-treated group, but not in the liver cross-sections from the Exo-SrIκB-treated group (Figure 3B). The histopathological examinations revealed that the Exo-SrIκB treatment group showed a decrease in piecemeal necrosis (46%), lobular necrosis (25%), histological activity score (33%), liver fibrosis (34%), and total score (35%) compared with the vehicle-treated group (Figure 4A). The Masson’s trichrome staining results showed a 38.3% decrease in the degree of fibrosis in the Exo-SrIκB-treated group compared to the vehicle-treated group, correlating with the histopathological fibrosis speculum results (Figure 4B). Collectively, the results indicated that the administration of Exo-SrIκB resulted in a reduction in jaundice and the amelioration of inflammation and liver fibrosis compared with administration of the vehicle. Based on these results, the therapeutic effects of Exo-srIκB were confirmed in a porcine RFA-induced biliary stricture liver fibrosis model.

## 4. Discussion

The current therapeutic approach of biliary atresia involves improvement of symptoms through surgical intervention to release the blockage in the biliary tract and allow bile juice to move to the small intestine through the bile duct. Although some treatments such as glucocorticoids, antibiotics, and choleretic agents have been used as adjunctive therapies following portoenterostomy, the pharmacological treatment options for biliary atresia are very limited [35,36]. Both BDL-mediated liver fibrosis and RFA-induced biliary stricture are well-established animal models that successfully mimic the progression of biliary atresia and primary biliary cholangitis [37,38]. Therefore, those animal models can provide valuable insights into the therapeutic potential of treatments for biliary strictures and obstructions.

Recent breakthroughs in nanotechnology applications have enabled the development of nanomedicines for small-molecule therapies [28]. Exosome-based drug delivery offers several advantages due to its unique characteristics, which include high drug storage capacity, ability to bypass biological barriers, and targeted selectivity [21]. Regardless of the origin and size of the exosomes, distribution to the liver is observed within minutes, and its effects persist for several days, but can also be found in other organs, including the spleen and kidneys, indicating their therapeutic effects in various diseases [39,40,41,42], including chronic liver disease, renal ischemia-reperfusion injury, and sepsis-associated organ damage [27,28,43].

NF-κB has been recognized as a key regulator of inflammation in many hepatic cell populations but is also required for hepatocyte survival and liver homeostasis. The activation of the NF-κB signaling pathway leads to a severe increase in proinflammatory cytokines and tissue injury at different stages of cholestasis [44]. Exo-SrIκB, an engineered exosome carrying the IκB super-repressor protein, was designed for the treatment of both acute and chronic inflammatory conditions. In this study, we confirmed that the application of Exo-SrIκB effectively attenuated the progression of cholestasis-induced inflammation and liver fibrosis by inhibiting NF-κB nuclear translocation. Therefore, Exo-SrIκB was implicated as a potential therapeutic agent for the treatment of biliary stricture and malignant bile duct obstruction.

The primary active component of Exo-SrIκB, super repressor-IκB, has demonstrated efficacy in reducing inflammatory responses across various diseases by preventing the nuclear translocation of NF-κB [45]. It was also found that cholestasis-induced liver fibrosis was alleviated through the anti-inflammatory action of Exo-SrIκB. However, because NF-κB activation has a hepatoprotective role and inhibits apoptosis, in one study, liver injury was found to be reduced due to the increased activity of NF-κB in 3 day BDL mice [46]. According to previous studies, the Exo-srIκB mediated NF-κB regulation in alcoholic liver disease mouse model primarily occurs in Kupffer cells [28]. The analysis of cellular targeting using Cre exosomes revealed that, in the absence of surface engineering, Kupffer cells are the primary targets of exosomes rather than hepatocytes. Therefore, further research is warranted to elucidate the multifaceted roles of NF-κB activation and to address interspecies differences that may impact the translational potential of Exo-srIκB. Nevertheless, the experimental confirmation of Exo-SrIκB’s therapeutic effects in biliary atresia using different animal models provides critical insights for drug development. By effectively reducing fibrosis and inflammation in these models, Exo-SrIκB has shown its potential as a novel therapeutic agent. The application of Exo-SrIκB led to potent anti-inflammatory and antifibrotic properties in our rodent and minipig models, showing promise for the treatment of obstructive biliary diseases in humans. Since the present study focuses on the preventive effects of Exo-srIkB on liver fibrosis, future research will explore its therapeutic potential for other chronic liver diseases. Such studies will enhance the clinical applicability of Exo-srIkB as an intervention for intractable liver diseases.

## 5. Conclusions

Our study highlights the therapeutic potential of Exo-SrIκB as a novel approach for treating biliary atresia and other obstructive biliary diseases. By effectively inhibiting NF-κB nuclear translocation, Exo-SrIκB demonstrated potent anti-inflammatory and anti-fibrotic properties, leading to significant attenuation of cholestasis-induced liver fibrosis. The findings provide strong preclinical evidence that engineered exosomes can serve as promising drug delivery systems for targeting inflammatory pathways in chronic liver disease.

## Data Availability

Data is contained within the article or Supplementary Materials.

## Supplementary Materials

The following supporting information can be downloaded at: https://www.mdpi.com/article/10.3390/pharmaceutics17020264/s1, Figure S1. Intravascular Exo-SrIκB administration attenuates BDL-induced liver fibrosis in mouse model. Figure S2. Intravascular Exo-SrIκB administration attenuates BDL-induced liver fibrosis in mouse model. Figure S3. Administration of Exo-SrlκB attenuates RFA-induced biliary stricture porcine model. Figure S4. Administration of Exo-SrlκB attenuates RFA-induced biliary stricture porcine model.

## Abbreviations

NF-κB (Nuclear factor-κB), Exosome engineering for Protein Loading via Optically Reversible protein-protein interactions (EXPLOR), SrIκB (super repressor IκB), α-SMA (alpha-smooth muscle actin), BDL (bile duct ligation), RFA (Endobiliary radiofrequency ablation), SEC (Size Exclusion Chromatography), TFF (Tangential flow filtration), ALT (alanine transferase), AST (aspartate transferase), ALP (alkaline phosphatase), and GGT (gamma glutamyl transferase).
