# Supplementary material for: Engineered Exosomes Carrying Super-Repressor IκB Reduced Biliary Atresia-Induced Liver Fibrosis in Minipig and Mouse Models
Source: Pharmaceutics. 2025 Feb 17;17(2):264. doi: 10.3390/pharmaceutics17020264 (PMC11859306; doi:10.3390/pharmaceutics17020264)
Supplement: Supplementary file 1 [file pharmaceutics-17-00264-s001.zip › pharmaceutics-3396225-supplementary.pdf]

Supplementary Materials

Engineered Exosomes Carrying Super-Repressor IκB Reduced Biliary Atresia-Induced Liver Fibrosis in Minipig and Mouse Models

Jisoo Kang, Cheolhyoung Park, Hanoul Yun, Chulhee Choi and Wonhyo Seo

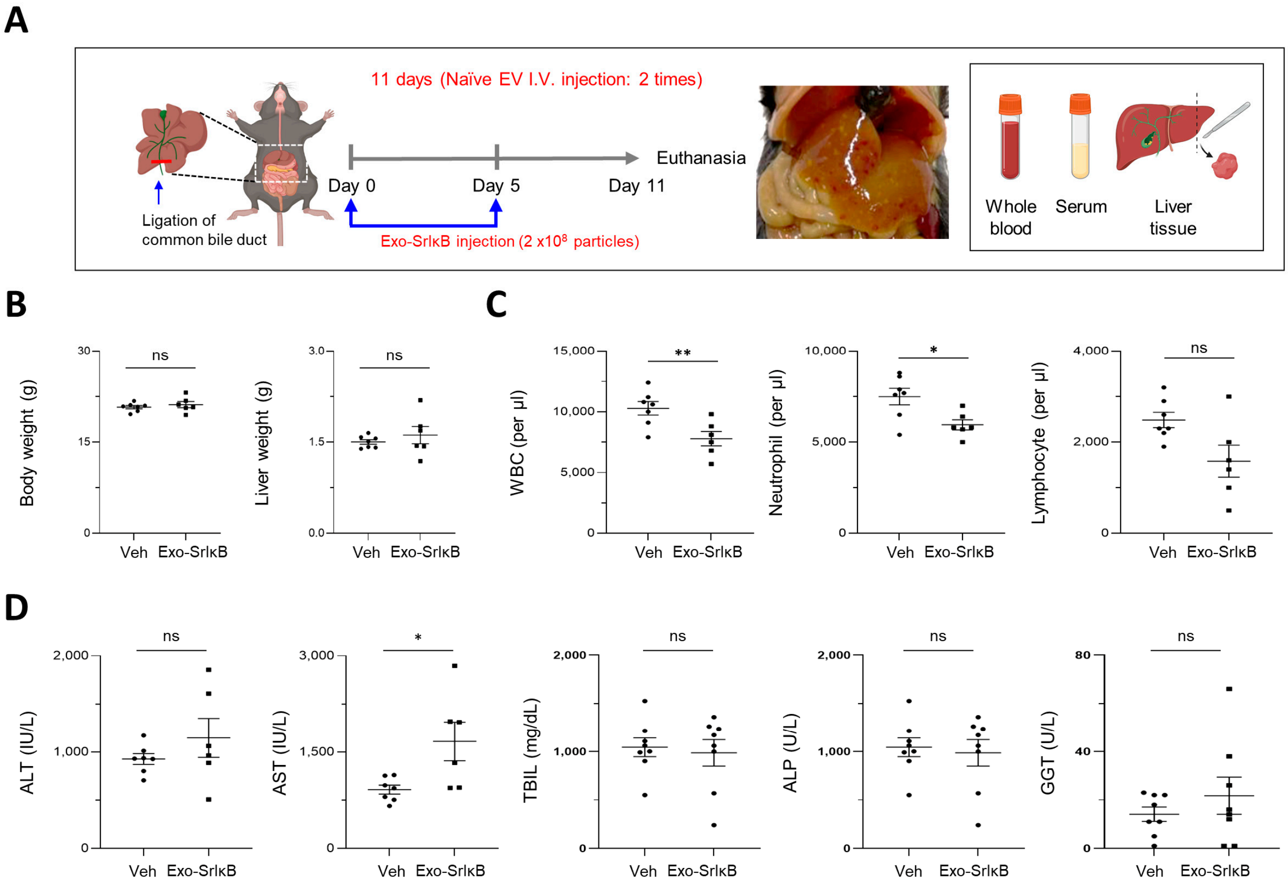

Figure S1. Intravascular Exo-SrIκB administration attenuates BDL-induced liver fibrosis in mouse model. (A) Schematic overview of the experimental design. (B) Body and liver weights were measured at euthanasia. (C, D) Whole blood and serum were subjected to complete blood counts and serum chemistry analyses. Data are expressed as the mean ± SEM. *$P < 0.05$, **$P < 0.01$ versus the corresponding control.

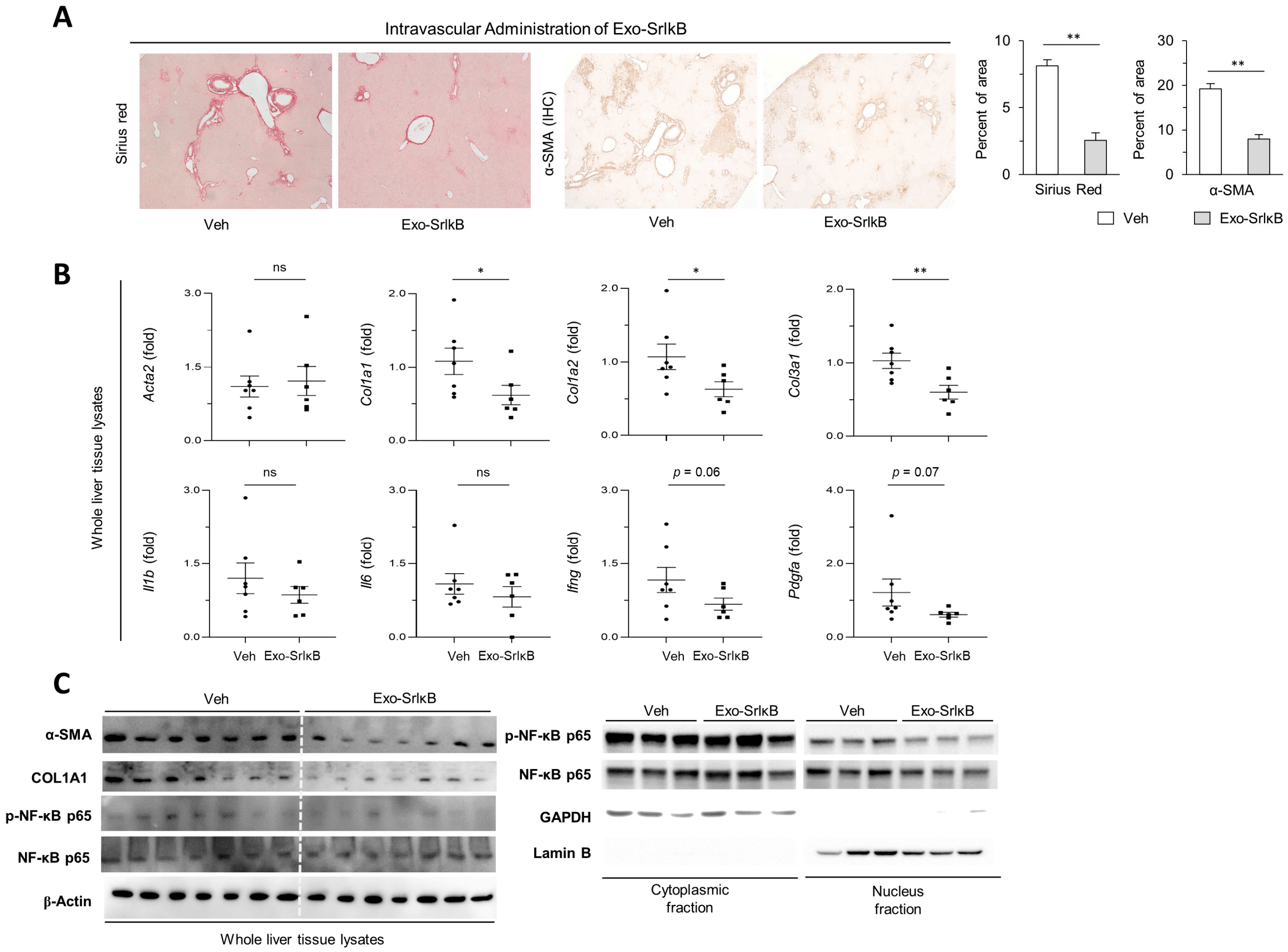

Figure S2. Intravascular Exo-SrIκB administration attenuates BDL-induced liver fibrosis in mouse model. (A) Liver tissues were subjected to Sirius staining, and α-SMA IHC staining. (B) Liver tissues were subjected to RT-qPCR analyses. (C) Protein expression (whole liver lysates and cytoplasmic/nucleus fractions) was assessed by immunoblotting. Data are expressed as the mean ± SEM. *$P < 0.05$, **$P < 0.01$ versus the corresponding control.

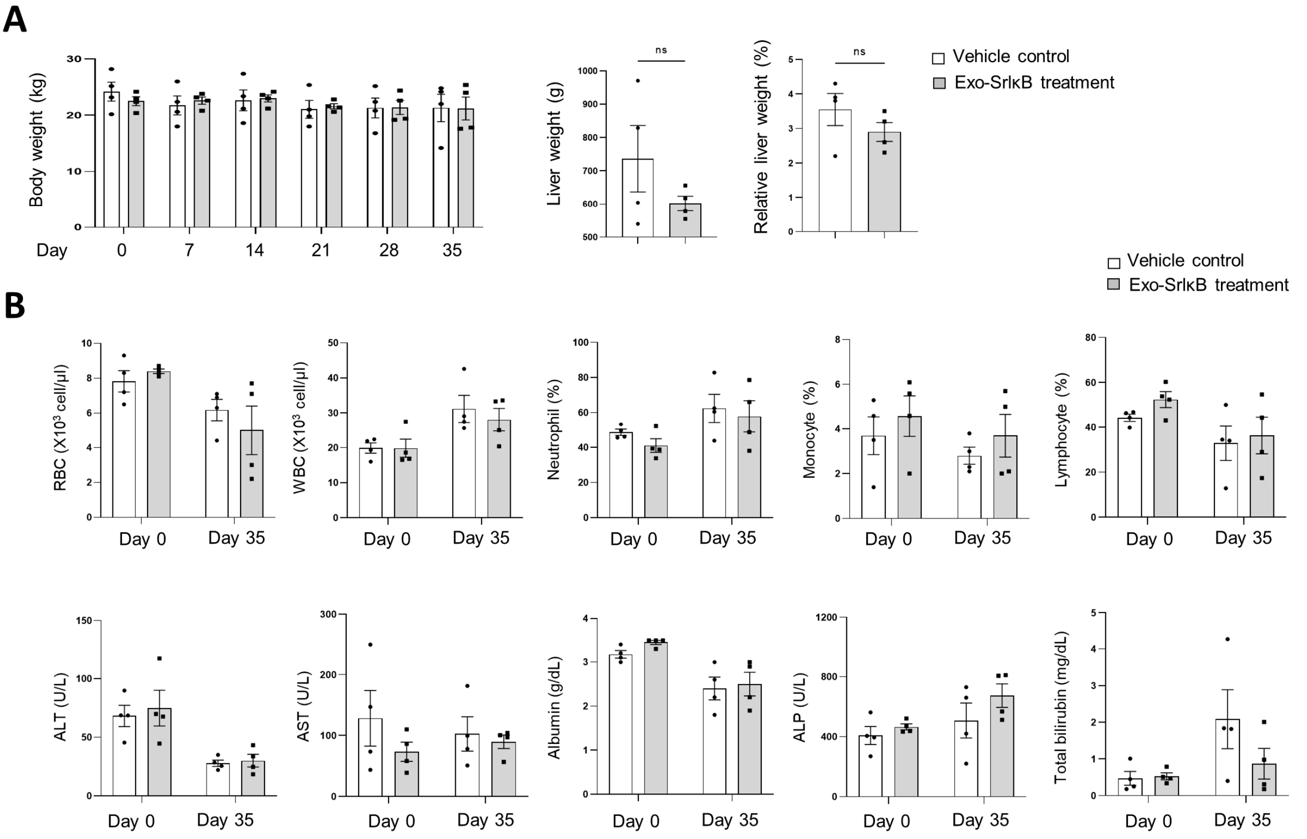

Figure S3. Administration of Exo-SrIκB attenuates RFA-induced biliary stricture porcine model. (A) The body weight, liver weight and relative liver weight were assessed. (B) Whole bloods were collected at several timelines (Day 0 and Day 35) during experiments. Analysis of complete blood count and serum chemistry were evaluated.

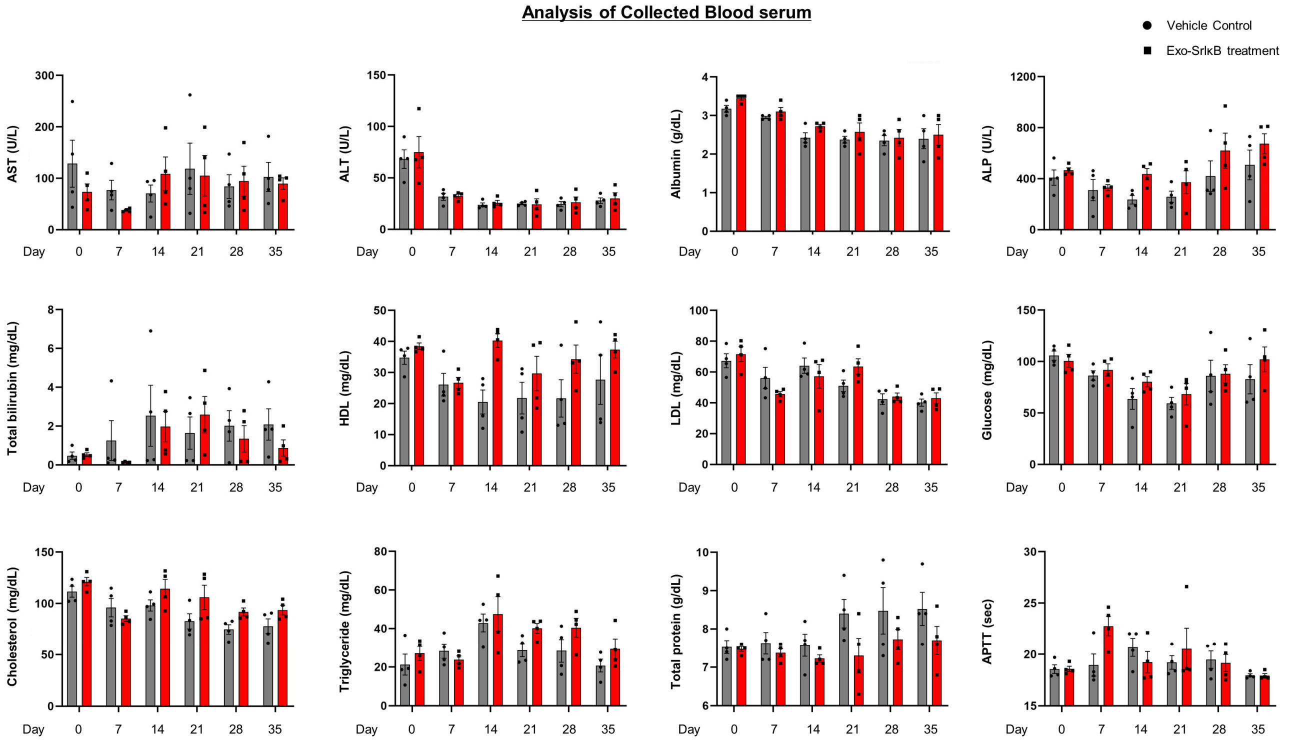

Figure S4. Administration of Exo-SrIκB attenuates RFA-induced biliary stricture porcine model. Collected sera were subjected to serum chemistry analyzer at several timelines during experiments.

AST: Aspartate Aminotransferase; ALT: Alanine Aminotransferase; ALP: Alkaline Phosphatase; HDL: high-density lipoprotein; LDL: low-density lipoprotein; APTT: Activated Partial Thromboplastin Time.
